# Age-related influence on DNA damage, proteomic inflammatory markers and oxidative stress in hospitalized COVID-19 patients compared to healthy controls

**DOI:** 10.1016/j.redox.2023.102914

**Published:** 2023-10-03

**Authors:** Agnes Draxler, Amelie Blaschke, Jessica Binar, Maria Weber, Michael Haslacher, Viktoria Bartak, Laura Bragagna, George Mare, Lina Maqboul, Rebecca Klapp, Theresa Herzog, Marton Széll, Agnese Petrera, Brenda Laky, Karl-Heinz Wagner, Rainer Thell

**Affiliations:** a Department of Nutritional Sciences, University of Vienna, Austria; b Research Platform Active Ageing, University of Vienna, Austria; c Vienna Doctoral School for Pharmaceutical, Nutritional and Sport Sciences (PhaNuSpo), University of Vienna, Josef Holaubek-Platz 2, 1090, Vienna, Austria; d Medical University of Vienna, Austria; e Klinik Donaustadt, Emergency Department, Langobardenstraße 122, 1220, Vienna, Austria; f Metabolomics and Proteomics Core, Helmholtz Zentrum München, German Research Center for Environmental Health (GmbH), Neuherberg, Germany; g Austrian Society of Regenerative Medicine, Vienna, Austria

**Keywords:** SARS-COV-2, COVID-19, DNA damage, Oxidative stress, Inflammation, Hospitalized patients, Proteomics

## Abstract

COVID-19 infections are accompanied by adverse changes in inflammatory pathways that are also partly influenced by increased oxidative stress and might result in elevated DNA damage. The aim of this case-control study was to examine whether COVID-19 patients show differences in oxidative stress-related markers, unconjugated bilirubin (UCB), an inflammation panel and DNA damage compared to healthy, age-and sex-matched controls.

The Comet assay with and without the treatment of formamidopyrimidine DNA glycosylase (FPG) and H_2_O_2_ challenge was used to detect DNA damage in whole blood. qPCR was applied for gene expression, UCB was analyzed via HPLC, targeted proteomics were applied using Olink® inflammation panel and various oxidative stress as well as clinical biochemistry markers were analyzed in plasma.

Hospitalized COVID-19 patients (n = 48) demonstrated higher serum levels of 55 inflammatory proteins (p < 0.001), including hs-C-reactive protein levels (p < 0.05), compared to healthy controls (n = 48). Interestingly, significantly increased age-related DNA damage (%-DNA in tail) after formamidopyrimidine DNA glycosylase (FPG) treatment was measured in younger (n = 24, average age 55.7 years; p < 0.05) but not in older COVID-19 patients (n = 24, average age 83.5 years; p > 0.05). Although various oxidative stress markers were not altered (e.g., FRAP, malondialdehyde, p > 0.05), a significant increased ratio of oxidized to reduced glutathione was detected in COVID-19 patients compared to healthy controls (p < 0.05). UCB levels were significantly lower in individuals with COVID-19, especially in younger COVID-19 patients (p < 0.05).

These results suggest that COVID-19 infections exert effects on DNA damage related to age in hospitalized COVID-19 patients that might be driven by changes in inflammatory pathways but are not altered by oxidative stress parameters.

## 1 Introduction

The outbreak of the coronavirus pandemic starting in early 2020 impaired health systems dramatically and pushed hospitals’ treatment capacities to the edge worldwide. The severe acute respiratory coronavirus 2 (SARS-CoV-2) is the pathogenic virus causing coronavirus disease (COVID-19) in humans. COVID-19 symptoms are diverse, ranging from mild to severe, sometimes with lethal outcomes. As described by Fauci et al. [1] mortality risk as well as the severity of an ongoing COVID-19 infection are associated with older age. Current data suggest that comorbidities including diabetes mellitus type 2 (T2DM), obesity as well as hypertension increases mortality risk manifolds, especially in people of advanced age [2,3]. These underlying conditions are known risk factors that might shape the progression and severity of COVID-19 symptoms during an ongoing infection. Strikingly, genomic instability is also associated with various diseases such as cancer, T2DM, or generally aging [4,5], since older age is linked to low-grade chronic inflammation, which seems to be a pervasive feature accompanied by potentially declining responses of the innate immune system. Further, prominent elevations of key inflammatory mediators including interleukin-6 (IL-6), or C-reactive protein (CRP) along with immunosenescence can be observed in older adults [[6], [7], [8]]. Chronic low-grade inflammation in older individuals can be further driven by increased levels of oxidative stress and reduced T-cells activation that might contribute to greater severity of virus-induced infections. Studies reported that corona-virus derived protein deposits can lead to elevated oxidative stress in the endoplasmic reticulum and can be accompanied by mitochondrial dysfunction in virus-affected epithelial cells [9]. Thus, the presence of viral-derived proteins can not only lead to suppression of receptor-based induced innate immune responses such as the toll-like receptors (TLR), but also to an independent activation of the inflammasome stimulated by the stress-induced production of reactive oxygen species (ROS). As mentioned, the interplay of oxidative stress and inflammation might play a potential role in the pathogenesis during a SARS-CoV-2 infection. For example, studies in middle-aged mice have shown that lipid peroxidation caused by chronic oxidative stress increases the expression of the Phospholipase A2 Group IID (PLA2G2D) which are precursors of the secretory phospholipase A2. This increase in PLA2G2D expression was associated with reduced survival as well as impaired T-cell responses as a consequence of a SARS-CoV-1 infection [10]. An imbalanced production of ROS results in harmful oxidative stress that might promote chronic diseases such as cancer or cardiovascular disease [11]. In general, viral infections are known to promote a pro-oxidative milieu in the cellular environment of the host in order to maintain the viral replication cycle [12,13]. One in vitro study investigated the function of glutathione in SARS-CoV-2 infected Vero E6 cells and glutathione depletion could be detected in these cells by reducing the levels of thiols, particularly reduced glutathione (γ-Glutamyl-Cysteinyl-Glycin, GSH) and thereby disturbing redox balance of cellular thiols [14]. However, studies investigating glutathione status in SARS-Cov-2 infected patients are scarce. Studies suggest that viral infections might also contribute to a higher risk of genomic instability, thus elevated cancer risk. Different viral infections are known to be linked to an increased risk of certain types of cancer in humans [15]. For example, the human papillomavirus (HPV) are prominent carcinogenic viruses that facilitate the production of oncogenic proteins E6 and E7 that in turn suppresses the activity of tumor suppressor proteins p53 and RB1 [15]. This inevitably leads to major disturbances in cell cycle activity by interfering with pivotal cellular events such as DNA repair and DNA replication. This might result in the formation of DNA lesions and single strand breaks that contribute to chromosome instability [16,17]. Maintaining DNA integrity is of utmost importance to reduce the risk of deleterious effects on genomic stability such as mutations [18]. One study suggests that there might be a link between higher levels of DNA damage in patients with severe pneumonia during COVID-19 infections [19]. Further, it has been shown that comorbidities such as hypertension play a decisive role when it comes to severity of COVID-19 infections [20]. It has been shown [21] that COVID-19 might induce oxidative stress as a result of the production of ROS in response to inflammatory processes, while others have reported that patients suffering from acute COVID-19 infections showed lower antioxidant capacity in contrast to healthy individuals [21]. However, it has also been demonstrated that antioxidant capacity might be improved after recovery from the infection [22].

Malondialdehyde (MDA) is an oxidant marker for lipoperoxidation [23], and the ferric reducing ability potential assay (FRAP) in plasma is a frequently used test to evaluate the antioxidant capacity [24]. The nuclear factor erythroid 2-related factor (NrF2) is capable of sensing oxidants and is a major regulator of the internal antioxidant defense system [25,26]. NrF2 is associated with numerous chronic diseases that are also related to oxidative stress such as cardiovascular disease, metabolic syndrome, T2DM, and cancer [11].

Liver injury and extreme elevations of bilirubin levels might also be associated with inflammation and increased mortality in COVID-19 patients [27]. However, slightly elevated levels of unconjugated bilirubin (UCB) stemming of the breakdown from hemoglobin are associated with positive health outcomes, including reduced body mass index (BMI), improved body composition, a higher antioxidant potential and even decreased risks for cardiovascular diseases [28]. Since mildly elevated levels of indirect bilirubin are also known to exert antioxidant properties in the blood, it might be protective against oxidative damage by exerting free radical scavenging properties [29,30]. This might also reduce the risk of oxidative damage. Recent studies have demonstrated that UCB levels can be affected during infections and disease [31]. Yet, no studies exist that investigated UCB in COVID-19 hospitalized patients compared to healthy age- and sex-matched controls.

Thus, the aim of this age-and gender-matched case-control study was to comprehensively investigate whether DNA damage, antioxidative and oxidative stress parameters as well as proteomic inflammatory markers differ between acutely infected COVID-19 patients compared to their healthy counterparts.

## 2 Material and methods

### 2.1 Study design and participants

**Fig. 1:**
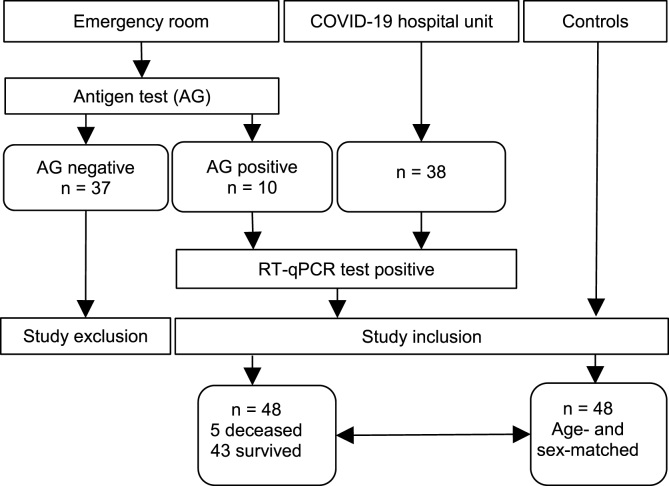
Flowchart of the ABCD-COVID-19 study.

The “Analysis Blood Covid DNA” (ABCD) study was a prospective case control study performed at the Department of Internal Medicine 2, Emergency Department, Clinic Donaustadt and the Department of Nutritional Sciences, University of Vienna. Recruitment of the COVID-19 patients (n = 48) was performed at the Emergency Department of the Clinic Donaustadt in the period of December 2020 to February 2021 (see Fig. 1). Age- and gender-matched controls (n = 48) were obtained from volunteers of university and hospital staff members and from a sample pool older adults from an ongoing projects (‘NutriAging Vitamin D’: https://clinicaltrials.gov/ct2/show/NCT04341818 and the ‘protein project’: https://clinicaltrials.gov/ct2/show/NCT04023513). None of the patients were vaccinated against SARS-COV-2 at the time of blood sampling and among the healthy controls only one person was vaccinated.

Inclusion criteria for COVID-19 patients were: gender male/female, age: ≥40 years, patients admitted at the emergency department, COVID-19 suspected patients, able to give written informed consent; Exclusion criteria for COVID-19 patients were: children (≤18 years) and adults 19–39 years, not hospitalized, but confirmed COVID-19 patients (outpatients), patients without definitive COVID-19 confirmation, pregnancy. Inclusion criteria for healthy controls were: gender male/female, age: ≥40 years, absence of severe illnesses/serious diseases, able to give written informed consent. Exclusion criteria for health controls were: children (≤18 years) and adults 19–39 years, any current or past clinically significant pathology/disease (comorbidity) pregnancy. The study was approved by the Ethical Commission of the City of Vienna (No. EK_20_284_1120) and was conducted in accordance with the approved guidelines by the Declaration of Helsinki. Written informed consent was received prior to participation. The study was registered at ClincalTrilals.gov (Identifier: NCT04784468).

### 2.2 Blood sampling and sample preparation

Blood samples were drawn from COVID-19 patients and healthy controls. After confirming an acute COVID-19 infection in patients using SARS-CoV-2 rapid antigen tests (Roche) combined with quantitative real time polymerase chain reaction (RT-qPCR), blood samples were taken after overnight fast from every patient using EDTA-plasma, serum tubes (BD Vacutainer®) and Paxgene® blood RNA collection tubes (BD Paxgene™). Samples were protected from light and immediately processed after blood collection as well as aliquoted for further analyses. Samples were stored at−80 °C and were kept cool after thawing until analysis.

### 2.3 Blood chemistry

After blood sampling, blood parameters including complete blood count of red and white blood cells, hemoglobin, hematocrit, glucose, triglycerides, total cholesterol, high density lipoprotein (HDL), low density lipoprotein (LDL), aspartate amino transferase (ASAT), alanine transaminase (ALAT), high sensitivity c-reactive protein (hs-CRP), uric acid, and creatinine plasma levels of COVID-19 patients were analyzed in the central laboratories of the Clinic Donaustadt. The same parameters were analyzed for the healthy control group in the routine laboratory of Dr. Claudia Vidotto (1230 Vienna, Austria). Vitamin D has been assessed using a commercially available ELISA Kit measuring 25-OH-vitamin D (Euroimmun Medical Lab Diagnostics, Austria) according to the manufacturers protocol. All healthy controls were tested for SARS-CoV-2 antibodies using a commercially available Anti-SARS-CoV-2 IgG ELISA Kit (Euroimmun Medical Lab Diagnostics, Austria).

### 2.4 Analyses of malondialdehyde, antioxidant capacity (FRAP) and glutathione as markers of oxidative stress

MDA levels were determined in plasma as described previously [32]. After heating (60 min, 100 °C), plasma samples were neutralized with methanol/NaOH, centrifuged (3 min, 3000 rpm) and their MDA was measured with HPLC (excitation: *λ* 532 nm, emission: *λ* 563 nm, LaChrom Merck Hitachi Chromatography System, Vienna, Austria; HPLC column 125 × 4 mm, 5 μm; Merck, Vienna, Austria). The antioxidant capacity of serum was measured via the ferric reducing ability potential (FRAP) assay as described earlier [24] in triplicates, using trolox as a standard. Absorbance was measured with BMG FLUOstar OPTIMA Microplate Reader (BMG LABTECH GmbH) at 593 nm and results are expressed as trolox equivalents in μmol/L. GSSG (glutathione disulfide) and GSH were analyzed with use of N-Ethylmaleimide and O-phthalaldehyde according to an adopted method of Hissin and Hilf [33] as described previously [34]. All samples were analyzed fluorometrically in triplicates with external standards of GSSG and GSH using BMG FLUOstar OPTIMA Microplate Reader (BMG LABTECH GmbH).

### 2.5 HPLC analyses of unconjugated bilirubin

Indirect bilirubin levels (UCB) were measured in plasma samples by performing high-performance liquid chromatography analyses (HPLC; Merck, Hitachi, LaChrom) according to a widely-used protocol [28,29,35]. For the analyses, a Fortis C18 HPLC column (4.6 × 150 mm, 3 μm), cartridges (Phenomenex SecurityGuard™), and a photodiode array detector (PDA, Shimadzu) were used. All plasma samples were centrifuged and 50 μl sample was mixed with the isocratic mobile phase consisting of glacial acid (6.01 g/L) as well as 0.1 M n-dioctylamine solution in HPLC-grade methanol/water (96.5/3.5%). The samples were centrifuged repeatedly in order to inject 120 μl of the supernatant into the HPLC (flow rate: 1 mL/min). To reduce batch-to-batch fluctuation, all case-control pairs were analyzed using the same plate run and bilirubin α (purity≥98%, Sigma Aldrich) was used as the external standard.

### 2.6 Assessment of DNA damage

DNA damage in whole blood was assessed with the Comet assay, a method for visualizing DNA damage in single cells. The protocol was performed as already described by Draxler et al. [36]. 10 μl of whole blood samples were mixed with 200 μL 1%-agarose solution and 5 μL of the mixture were spotted onto 12 spot microscopy slides. For every blood sample, 4 slides were needed due to 4 different treatments. To purify the DNA of the blood samples from cell debris all microscopy slides were treated with a lysis buffer for 1 h. Two of the microscopy slides were washed 3 times with enzyme buffer (40 mM HEPES, 0.1 M KCL, 0.5 mM EDTA, 0.2 mg/mL BSA, pH 8). On each spot of one of the two slides 30 μl of the lesion-specific enzyme FPG (diluted 1:3500 in enzyme buffer) was applied, using a slide unit (12-Gel Comet Assay Unit™, Severn Biotech Limited, Kidderminster, UK). On the second slide 30 μl of the enzyme buffer alone was applied. Both slides were incubated at 37 for 30 min. Treatment with enzyme buffer alone acted as a blank for the FPG treatment. The fourth slide was incubated for 15 min with the oxidizing agent H₂O₂ (100 μM), which allowed indirect measurement of antioxidant status and represented resistance to H₂O₂. After all treatment steps the DNA on the microscopy slides had to unwind in an alkaline electrophoresis puffer for 20min (0.3 M NaOH, 1 mM EDTA, pH 13), followed by a 30-min electrophoresis run (25 V, 300 mA, 4 °C). The extent of DNA damage could then be visualized and evaluated by coloring the DNA with GelRed (GelRed TM Nucleic Acid Gel Stain, VWR, product no. 41003) and using a fluorescence microscope (Nikon) and the software Comet Assay IV was used to accurately determine DNA damage.

### 2.7 Analyzing inflammation using OLINK proteomics panel

The expression of 92 inflammation-related protein biomarkers in plasma were analyzed by the Target96 Inflammation Panel (version 95302) from Olink® Biosciences, Uppsala, Sweden. Assay characteristics and validations are available from the manufacturer's webpage at https://www.olink.com/question/are-internal-controls-included-in-the-assay-and-if-sowhat-are-they). In brief, antibodies labelled with complementary oligonucleotide sequences could bind pairwise to the target protein. Upon DNA-hybridization, the paired oligonucleotides form a reporter sequence that was amplified by RT-qPCR. Data are expressed as Normalized Protein.

Expression (NPX) units on a log2 scale calculated from normalized Ct values. Samples that deviated more than 0.3 NPX from the median value of an internal control were excluded. The lower limit of detection (LOD) was defined as three standard deviations above background. 71 proteins with >75% of samples above LOD were included in the analyses and, in accordance with recommendations by the company, values below LOD were not replaced by arbitrary values field. Also, samples that were classified as flagged by Olink® were excluded from further analyses. The samples were prepared in Vienna (Austria) and sent on dry ice to Munich (Germany) for analysis. The Olink® R package (v.3.3.1.) was applied to evaluate protein abundance between COVID-19 vs. healthy controls, older COVID-19 hospital patients (>69 y) vs. healthy controls, younger COVID-19 hospital patients (<69 y) vs. healthy controls, younger COVID-19 hospital patients vs. older COVID-19 patients. Adjusted p-values <0.05 were accepted as statistically significant.

### 2.8 Gene expression analyses using qPCR

To analyze differences in gene expression, whole blood was drawn from the participants directly into 2.5 mL PAXgene™ blood RNA tubes. After storing the RNA tubes for 24h at room temperature, blood tubes were stored at −80 °C until processing according to the manufacturers protocol (Qiagen, CA). Quantity and purity of the RNA samples was assessed with the Nanodrop™ 2000 spectrophotometer (Thermo Fisher Scientific). In order to perform cDNA synthesis, a commercially available kit was used (High-capacity cDNA Reverse Transcription Kit, Applied Biosystems™, Thermo Fisher Scientific). qPCR was used in order to verify gene expression differences. For all qPCR runs, the 384-well QuantStudio™ 6 Flex Real-Time PCR System (Applied Biosystems™, Thermo Fisher Scientific) and a SYBR green-based mastermix (SYBR® Select Master Mix, Applied Biosystems™ Thermo Fisher Scientific) was used according to the manufacturers' protocol. All primers were tested for target gene specificity using NCBI primer blast and were ordered from Sigma-Aldrich in a lyophilized state. Primers were then dissolved in Nuclease-Free Water to produce 100 μM primer stock solutions and were diluted to 16 μM working solutions. After pretesting each primer pair, one 384-well plate was used for each gene to analyze all the samples at once in order to minimize inter-plate variations. Primer for the reference genes were selected based on NormFinder® analyses. Quality criteria were following the MIQE guidelines for RNA extraction and analyses and Relative gene expression was calculated according to the 2^- ΔΔ^^Ct^ method by Livak et al. [37].

### 2.9 Statistics

Statistical analyses were performed using IBM SPSS Statistics 28. Normal distribution was analyzed with the Kolmogorov-Smirnov test. Comparison of COVID-19 patients and control individuals was performed using the independent samples *t*-test (parametric data) or Mann–Whitney *U* test (non-parametric data) restricted to participants with no missing data. Correlations between variables were analyzed by Pearson or Spearman correlation. Gene expression was analyzed in a logarithmic scale by calculating the log2fold change of the ratio of gene expression levels between samples from COVID-19 patients compared to their healthy counterparts by using Mann-Whitney *U* test. A p-value of 0.05 is considered significant.

## 3 Results

### 3.1 Study cohorts

A total of forty-eight COVID-19 patients (average age of 69.3 ± 16.7 years) and 48 healthy (23 females and 25 males) sex- and age-matched controls (average age of 69.4 ± 16.5 years were enrolled in the study (Table 2). Acute symptoms caused by the COVID-19 infection and comorbidities of the COVID-19 patient cohort are presented in Table 1 and Table 3, respectively. Individuals with COVID-19 displayed an average Ct-value of 25.23 ± 0.47.

**Table 1:** Acute symptoms caused by the COVID-19 infection.

	All		Females		Males	
	n = 48	%	n = 23	%	n = 25	%
**COVID-19 symptoms**						
Shortness of breath	22	22.9	9	39.1	13	52.0
Fatigue	22	22.9	9	39.1	13	52.0
Cough	21	21.9	9	39.1	12	48.0
GI issues	17	17.7	9	39.1	8	32.0
Chest pain	11	11.5	7	30.4	4	16.0
Dizziness	10	10.4	5	21.7	5	20.0
Headache	9	9.4	3	13.0	6	24.0
Hypotony	8	7.3	4	16.0	4	16.0
Sensory loss	8	8.3	4	17.4	4	16.0
Appetite loss	5	5.2	4	17.4	1	4.0
Joint ache	5	5.2	1	4.3	4	16.0

GI: Gastrointestinal issues (Nausea, Diarrhea).

**Table 2:** Demographic characteristics of study participants: Healthy vs. COVID-19 hospital patients.

Parameter	Controls	COVID-19 patients	p-value
Subjects	n = 48	n = 48	
	mean ± SD	mean ± SD	
Age (years)	69.4 ± 16.5	69.3 ± 16.7	
Sex (females/males)	23/25	23/25	
**DNA damage parameters**			
Lysis (%)	1.78 ± 0.54	2.03 ± 0.74	0.070
Netto FPG (% Tail DNA)	7.86 ± 2.34	9.21 ± 5.11	0.106
H_2_O_2_ (% Tail DNA)	11.44 ± 2.82	12.44 ± 2.90	0.083
**Oxidative stress parameters**			
FRAP (μmol/L)	982 ± 228	956 ± 256	0.374
GSH (μmol/L)	15.14 ± 2.96	14.35 ± 3.73	0.061
GSSG (μmol/L)	10.26 ± 2.58	8.17 ± 3.80	**<0.001**
GSH/GSSG ratio	1.57 ± 0.54	1.96 ± 0.79	**0.004**
MDA (μmol/L)	1.55 ± 0.40	1.56 ± 0.58	0.629
**Blood parameters**			
Erythrocytes (T/L)	4.88 ± 1.15	4.33 ± 0.69	**0.005**
Leucocytes (G/L)	6.33 ± 1.60	6.71 ± 3.53	0.498
Lymphocytes (G/L)	1.98 ± 0.55	1.29 ± 1.28	**<0.001**
hs-CRP (mg/L)	2.00 ± 2.34	76.88 ± 72.04	**<0.001**
HDL (mg/dL)	63.00 ± 15.26	33.19 ± 11.99	**<0.001**
LDL (mg/dL)	144.50 ± 43.61	81.56 ± 35.14	**<0.001**
Total cholesterol (mg/dL)	229.40 ± 46.00	136.6 ± 40.22	**<0.001**
Blood glucose (mg/dL)	104.40 ± 26.20	128.1 ± 68.76	**0.029**
ALAT (U/L)	27.44 ± 25.09	38.49 ± 35.93	0.093
ASAT (U/L)	26.69 ± 13.83	40.88 ± 27.34	**0.030**
UCB (μmol/L)	3.80 ± 2.81	2.63 ± 3.11	**<0.001**
Vitamin D serum level (ng/mL)	22.6 ± 12.5	22.4 ± 9.2	0.755

Data are presented as mean ± standard deviation, p-values are calculated using Mann-Whitney *U* test for measuring gender differences, significant differences are highlighted with bold numbers. Controls and COVID-19 hospital patients are age and sex-matched. FPG: formamidopyrimidine DNA glycosylase, FRAP: ferric reducing ability potential; GSH:γ-glutamyl-cysteinyl-glycine; GSSG: glutathione disulfide; MDA: malondialdehyde; hs-CRP: high sensitivity c-reactive protein; ALAT: alanine-minotransferase; ASAT: aspartate-aminotransferase, UCB: unconjugated bilirubin; HDL: high density lipoprotein; LDL: low density lipoprotein.

**Table 3:** Comorbidities of the COVID-19 patients.

	All		Females		Males	
	n = 48	%	n = 23	%	n = 25	%
**Comorbidities**						
Hypertension	25	52.1	15	65.2	10	40.0
T2DM	14	29.2	8	34.8	6	24.0
CHD	6	12.5	3	13.0	3	12.0
MCI	4	8.3	1	4.3	3	12.0
DVT	3	6.3	1	4.3	2	8.0
Allergies	3	6.3	2	8.7	1	4.0
Respiratory diseases*	3	6.3	2	8.7	1	4.0
Smokers	3	4.2	2	8.7	1	4.0
Insult	2	4.2	1	4.3	1	4.0
Cancer survivor	1	2.1	1	4.3	0	0
Other illnesses**	6	7.3	1	4.3	5	20.0

T2DM: Type 2 diabetes mellitus; CHD: coronary heart disease;

MCI: mild cognitive impaired, DVT: deep venous thrombosis; *Asthma, COPD, **Gout, colonic ileus, lymphopenia, cirrhosis of the liver, sepsis.

Five male participants above the average age of 69 years passed away as a consequence of the COVID-19 infection. Subgroup analyses showed that they displayed significantly lower Ct-values when confirming COVID-19 infections via qPCR test (average mean of Ct-value 19 vs. average mean of survivors’ values: 25.6; p = 0.006). Creatinine values were significantly elevated (2 mg/dl vs. 1.1 mg/dl; p < 0.001) compared to the other COVID-19 patients.

### 3.2 Blood parameters

Plasma hs-CRP levels were significantly increased in COVID-19 patients (+3790%; p < 0.01). They also showed elevated levels of liver enzymes such as ALAT (+40%) as well as ASAT (+53%) compared to healthy controls (p < 0.01). COVID-19 patients showed higher blood glucose levels (+23%, p < 0.05) and lower HDL-cholesterol (−48%, p < 0.01). The COVID-19 group even displayed lower total cholesterol (−40%, p < 0.01) and LDL-cholesterol (−44%, p < 0.01) plasma levels. Further, a significantly lower red and white blood cell count could be detected in COVID-19 patients, as outlined in Table 2. No differences in vitamin D levels could be detected between the study groups and both groups displayed an average plasma level of 22.5 ng/ml.

### 3.3 Oxidative stress marker and DNA damage parameters

As indicated in Table 2, there was no significant variation in the plasma levels of FRAP and MDA between COVID-19 infected patients and their matched controls. However, slightly lower GSH levels in individuals with COVID-19 could be observed without statistical difference (p = 0.062). Levels of GSSG and the ratio of GSH:GSSG were significantly lower in COVID-19 patients (p < 0.01, Table 2). Individuals with COVID-19 demonstrated significantly lower UCB levels (p < 0.01, Table 2).

DNA strand breaks (%-DNA in tail) showed no significant differences after treating the samples with lysis buffer or H_2_O_2_. Further, FPG sensitive sites were not different between the study groups. However, the analyses of age-related subgroups (age group 1: <69 years, age group 2: ≥69 years) revealed that in the younger COVID-19 patient group, significantly higher DNA strand breaks in FPG sensitive sites when compared to the healthy controls. There were no significant differences in DNA damage parameters in the older subgroups (see Table 4). The comparison between younger COVID-19 patients (average age of 55.7 ± 11.1 years; 11 females, 13 males) and the older COVID-19 subgroup (mean age 83.0 ± 7.1 years, 12 females, 12 males) revealed that significant differences in the FPG-induced DNA damage could be observed. Further, no significant difference regarding levels of DNA damage parameters (lysis, H_2_O_2_) was found between younger and older individuals affected by COVID-19 or between the healthy younger and older counterparts (Table 4 Supplementary). UCB was significantly higher in the younger COVID-19 group (p < 0.001). There was no difference in neither of the subgroups regarding MDA and FRAP plasma levels. As can be seen in Table 4, the comparison of younger COVID-19 patients with their healthy counterparts revealed significantly elevated levels of DNA damage after standard treatment with lyses buffer as well as H_2_O_2_. Regarding oxidative stress parameters, the GSH:GSSG ratio was significantly higher (p = 0.037) in younger COVID-19 patients compared with healthy controls, whereas UCB was significantly reduced in the younger COVID-19 group (Table 4). Sex-related subgroup analyses showed that female-COVID19 patients displayed significantly elevated DNA damage (p = 0.023) compared to their healthy controls after the basic treatment with lysis buffer. In male COVID-19 patients, no significant changes but tendencies of increased %-DNA damage could be observed for all challenges (lysis, FPG, H_2_O_2_) (Table 1 Supplementary).

**Table 4:** Parameters of COVID-19 hospital patients divided by age: 69 years.

Parameter	older COVID-19 patients (≥69 y)		p-value	younger COVID-19 patients (<69 y)		p-value
	Controls	COVID-19		Controls	COVID-19	
Subjects	n = 24	n = 24		n = 24	n = 24	
**DNA damage parameters**						
Lysis (% Tail DNA)	1.80 ± 0.46	1.89 ± 0.60	0.702	1.75 ± 0.62	2.17 ± 0.85	**0.048**
Netto FPG (% Tail DNA)	7.59 ± 1.90	7.67 ± 2.36	0.898	8.12 ± 2.73	10.69 ± 6.50	0.055
H_2_O_2_ (% Tail DNA)	11.87 ± 2.93	12.51 ± 3.43	0.686	11.02 ± 2.70	12.37 ± 2.35	**0.035**
**Oxidative stress parameters**						
FRAP (μmol/L)	989 ± 218	995 ± 319	0.684	976 ± 241	917 ± 199	0.452
GSH (μmol/L)	14.27 ± 2.84	13.81 ± 2.50	0.585	16.01 ± 2.88	14.91 ± 4.68	0.051
GSSG (μmol/L)	9.33 ± 2.60	7.84 ± 2.43	**0.026**	11.20 ± 2.23	8.52 ± 4.87	**<0.001**
GSH/GSSG ratio	1.68 ± 0.66	1.89 ± 0.59	0.127	1.47 ± 0.37	2.20 ± 1.65	**0.037**
MDA (μmol/L)	1.52 ± 0.36	1.50 ± 0.54	0.621	1.58 ± 0.44	1.61 ± 0.62	0.853
**Blood parameters**						
hs-CRP (mg/L)	1.93 ± 2.70	74.99 ± 75.28	**<0.001**	2.08 ± 1,97	78.94 ± 70.03	**<0.001**
UCB (μmol/L)	3.89 ± 3.49	3.10 ± 4.18	0.234	3.72 ± 2.12	2.20 ± 1.65	**<0.001**
Vitamin D (ng/ml)	23.00 ± 6.95	23.45 ± 12.62	0.984	21.72 ± 11.14	21.75 ± 12.50	0.711

Data are presented as means ± standard deviation, p-values are calculated using Mann-Whitney *U* test for measuring age differences, significant differences are highlighted with bold numbers. A p-value (p) of 0.05 is considered as significant. Older healthy and older COVID-19 hospital patients are age and sex-matched. FPG: ormamidopyrimidine DNA glycosylase, FRAP: ferric rducing bility potential; GSH: γ-glutamyl-cysteinyl-glycine; GSSG: glutathione disulfide; MDA: malondialdehyde; hs-CRP: igh ensitivity c-reactive protein; ALAT: alanine-minotransferase; ASAT: aspartate-aminotransferase, UCB: nconjugated ilirubin; HDL: igh density lipoprotein; LDL: olow density lipoprotein.

Independent of sex, significantly reduced levels of GSSG in COVID-19 patients could be detected compared to healthy controls. Further, the control groups displayed significantly reduced levels of GSH:GSSG ratios compared with male or female COVID-19 patients.

UCB was increased in COVID-19 females compared to their healthy controls but lower in males compared to their healthy controls (p < 0.05). Hs-CRP was significantly higher in COVID-19 patients in the total group as well as in all sex and age subgroups (p < 0.001, Table 2, Tables 1–3 Supplementary).

### 3.4 Proteomic inflammatory analyses

Sixteen of 92 protein biomarkers did not pass the QC criteria; therefore 76 proteins were considered in the statistical evaluation. Sixty-four inflammatory proteins out of the 76 proteins were significantly differently abundant in COVID-19 patients, whereas 55 proteins were significantly higher in the patients. The proinflammatory proteins with the highest expressions in COVID-19 patients included: CXCL11, PD-L1, TNF, IL6, MCP3, CXL10, C XCL23, IL6 and IL8; p < 0.05 (Fig. 3A). Nine proteins were significantly lower expressed that included CD6, SCF, CXCL5, TRANCE, Flt3L, DNER; p < 0.05. The results of the pathway analyses revealed that the inflammatory proteins that were found to be significantly expressed are associated with biological processes that are mainly involved in apoptotic processes, regulation of immune response, inflammatory response, chemotaxis, or secretion.

**Fig. 2:**
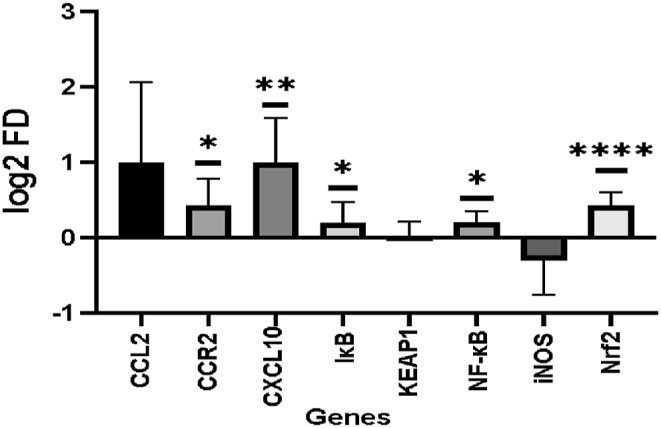
Difference in gene expression depicted as log2 fold change values of CCL2, CCR2, CXCL10, I-κB, KEAP1, NF-*κ*b, iNOS, Nrf2.The bars exhibit the log2 fold change expression of COVID-19 patients compared to healthy controls with standard deviation. Significant difference was analyzed using Mann-Whitney Test: The asterisk (*) indicates statistically significant difference. ns p > 0.05 *p ≤ 0.05 **p ≤ 0.01 ****p ≤ 0.0001.

**Fig. 3:**
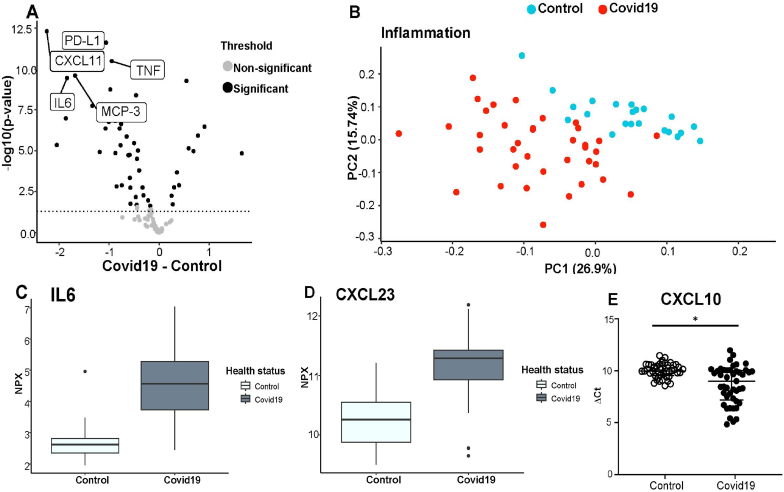
**A.** Volcano plot showing the significantly increased proteins and the he top 5 highly expressed inflammatory proteins detected in Covid19 patients using targeted proteomics analyses are depicted: PD-L1, CXCL11, TNF, IL6, MCP-3. The horizontal dotted line represents the threshold, grey dots below are non-significantly differently abundant proteins and the black dots above represent significantly differently abundant proteins. **B.** PCA plot shows visible clustering of inflammatory proteins between COVID-19-patients (red) compared to healthy controls (blue) The low overlap between the groups suggests that each group has unique inflammatory proteins expression profile. **C and D**: Boxplot showing the normalized protein expression (NPX) of the selected upregulated proteins (***, p < 0.001): **C.** IL6 **D**. CXCL23. **E**. Dot plot presentation of the Δ Ct showing higher expressed CXCL10 genes in COVID-19 patients vs. controls using qPCR also confirming the results obtained from proteomic analyses. Horizontal bars showing means ± standard deviation. The asterisk (*) indicates statistically significant difference.

Subgroup analyses based on sex showed that female COVID-19 patients expressed significantly higher levels of inflammatory proteins such as IL7, IL10RA, IL12B, IL17A, CCL4, CXCL6, FG23 and MMP10, compared to the female controls. However, CD6 was found to be lower in female COVID-19 patients. In male individuals suffering from COVID-19 it was observed that five inflammatory proteins were significantly higher abundant: OPG, AXIN1, CXCL1, CCL11 and CCL10. Subgroup analyses of the five deceased COVID-19 patients (all males) revealed significantly upregulated proteins such as CD8A, SLAMF1 and LIF-R, HGF, CCL25, NT3 compared to the surviving patients.

### 3.5 Gene expression analyses using qPCR

Gene expression differences between COVID-19 patients and healthy controls revealed that CCR2, CXCL10, I-κB, NF-κB and Nrf2 were significantly upregulated when analyzing the log2fold change expression values (Fig. 2), the iNOS gene showed tendencies. The KEAP1 gene showed no differences in gene expression between the two groups. CCL2, that is predominantly associated with viral and COVID-19 infections, was found to be upregulated in female and male patients (Fig. 1 Supplementary). Gene expression analyses of CCL2 indicated an upregulation in all COVID-19 patients and significantly different upregulations of CCL2 could be detected in females. It was evident that the distribution of the ΔCt-values of the CCL2 genewas broader in COVID-19 patients compared to healthy controls (Fig. 1 Supplementary).

### 3.6 Correlations of hs-CRP and age with DNA damage, oxidative stress and inflammatory parameters

COVID-19 patients showed significant correlations (all p < 0.01) of hs-CRP with IL8 (r = 0.421), MCP3 (r = 0.660), CXCL10 (r = 0.391), CCL23 (r = 0.555), and IL6 (r = 0.687). In healthy controls, there were no significant correlations detectable, except for IL-6 (r = 0.429; p < 0.01). When analyzing the COVID-19 patients age-related (cut-off median = 69 years) by dividing them into an older and younger subgroup, it has been shown that hs-CRP correlated significantly with MCP3 (r = 0.577), CCL23 (r = 0.542) and IL6 (r = 0.722, all p < 0.01) in younger COVID-19 patients. In contrast, young age and sex-matched healthy controls showed none of these correlations, except for IL6. However, in older COVID-19 patients more and stronger correlations could be detected with hs-CRP (all p < 0.01): IL8 (r = 0.548), MCP3 (r = 0.720), CXCL10 (r = 0.460), CCL23 (r = 0.577) and IL6 (r = 0.706). These correlations were not detectable in the healthy older cohort. In all case-control analyses and subgroup analyses, no significant correlations with parameters for DNA damage or oxidative stress could be observed.

Interestingly, DNA damage in FPG sensitive sites correlated negatively with age in the group of patients infected with COVID-19 (r = −0.336, p < 0.05). Age corelated with CXCL9 (r = 0.409) and VEGFA (r = 0.312, all p < 0.05) significantly but did not correlate with any other inflammatory parameters or parameter related to oxidative stress in COVID-19 patients. In the healthy cohort, a positive correlation between age and lysis treatment (r = 0.310, p < 0.05) and CXCL9 (r = 0.628, p < 0.05) could be detected.

## 4 Discussion

The aim of this case-control study, including 48 COVID-19 patients and 48 age-and gender-matched healthy controls, was to assess whether acute COVID-19 infections that required hospital treatment might be associated with age, elevated oxidative stress, and DNA damage as well as a large panel of proteomic inflammatory markers. Regarding DNA damage, we assessed a non-significant higher level in COVID-19 patients (Table 2) Especially after the incubation with lysis buffer and challenge with H_2_O_2_, DNA strand breaks (%-DNA in tail) showed higher values in the patients (+14%; p = 0.07 and + 8%; = 0.08, respectively). Additionally, damages in FPG-sensitive sites (oxidized purines) were even more pronounced in COVID-19 individuals (by +17%) compared to healthy controls (Table 2). This enzyme usually repairs oxidative DNA damage through cutting and removing 8-oxoguainin residues lesions from the DNA [38] indicating higher levels of oxidatively damaged DNA lesions in younger COVID-19 patients. When analyzing subgroups by age, we found some interesting age-related differences between younger and older COVID-19 patients (age group 1: <69 years, age group 2: ≥69 years). Challenges with H_2_O_2_ and lysis buffer did not show significant differences in DNA damage between the age groups. However, the younger COVID-19 cohort showed significantly higher FPG sensitive sites (by +39%) compared to older COVID-19 patients (Table 4 Supplementary). Thus, the enzymatic treatment, especially with FPG, enables the detection of oxidized purines such as 8-OH guanine and other ring-opened purine lesions [39] which are stronger pronounced in younger COVID-19 patients. Furthermore, the comparison of younger individuals with COVID-19 with their healthy controls showed significantly increased DNA damage after basic treatment with lysis buffer as well as after the challenge with H_2_O_2_ (p < 0.05) (Table 4). Unfortunately, five older male patients (aged above 69 years) enrolled in this study passed away due to the acute infection as a consequence of the COVID-19 infection. However, those men also suffered from underlying comorbidities. For example, three of the deceased patients had T2DM and/or hypertension, one individual suffered from coronary heart disease, while one person had asthma. They showed significantly lower Ct-values of 19 compared to 25.6 in the survivors, indicating a higher viral load [40] They also had significantly higher creatinine values (2 mg/dl vs. 1.1 mg/dl) which indicates impaired kidney functions. Also, other studies reported elevated DNA damage in COVID-19 patients [19,41] but did not detect age-related DNA damage during a COVID-19 infection. A recent publication by Gioia et al., 2023 [42] in human Huh-7 and Calu-3 cell lines showed that the SARS-Cov-2 specific proteins NSP13 and ORF6 are greatly involved in the initiation of DNA damage by degrading the DNA damage response kinase CHK1 in vitro. This, in turn, activates proinflammatory pathways demonstrating that COVID-19 infections contribute to DNA damage which increases inflammation in COVID-19 patients. As already reported in other studies [43], we also observed that COVID-19 patients had significantly highly expressed non-specific inflammatory markers such as hs-CRP levels in the plasma (Table 2) that might also induce the production of IL-6. Interestingly, Filbin et al. [44] conducted a longitudinal proteomic analysis in 306 COVID-19-positive patients and reported that COVID-19 patients with severe symptoms were younger (median age 58 years) compared to COVID-19 positive patients without symptoms (median age 67 years). In the mentioned study, it has been demonstrated that patients with severe COVID-19 symptoms displayed higher hs-CRP levels and ferritin levels compared to those lacking symptoms [44]. In our study, proteomic analysis (Olink assay) of individuals with COVID-19 also confirmed significantly increased protein abundance of IL-6 (Fig. 3C). As an acute inflammatory cytokine, IL-6 is involved in infections; especially in the lungs, and by exerting pyrogenic effects it plays an important role in the onset of infection-related fever [45,46]. Further, IL-6 upregulation has been reported to be gradually increased depending on COVID-19 disease severity in other studies [47] and with increased mortality [48]. Pathway analyses of the analyzed proteomic inflammatory markers (Fig. 2 Supplementary) revealed that those inflammatory proteins are linked to various biological processes such as apoptosis, immune and inflammatory responses, secretion, migration or chemotaxis. However, it should be noted that while proteomic analyses identified differences of numerous significant inflammatory proteins, these differences were relatively minor compared to the severely increased hs-CRP values found in COVID-19 individuals. Filbin et al. reported that the severity of the COVID-19 infections might shape the proteomic profile in plasma since COVID-19 patients with severe symptoms displayed higher expressions of proteins involved in the viral response and the IFN pathway including the proinflammatory cytokines such as CXCL10, CXCL11 as well as others involved into the response to vaccines, T cell function and innate immune activation [44]. Correlation analyses revealed that hs-CRP correlated significantly with certain proteomic parameters including IL-8, MCP3, or CXCL23 but not with oxidative stress parameters and DNA damage markers. Further, these correlations could not be observed in the healthy control group. Furthermore, no age-dependent differences in inflammatory markers were observed.

The liver enzyme ALAT was insignificantly higher in COVID-19 patients compared to the controls (+40.3%). ASAT levels were even significantly higher by 53% in the patients. Similar results regarding ASAT levels in COVID-19 patients have been documented by Basaran et al. [21]. Other studies have shown that elevated ASAT levels can be detected in diabetic animals and humans [49,50] and in hepatocytes of patients with chronic liver issues [49,51] that are also associated with DNA damage.

The gene expression analyses have confirmed that CXCL10 was upregulated in the COVID-19 patients compared to their sex-and age-matched healthy counterparts. NF-κB was upregulated in the COVID-19 patients (Fig. 2), while iNOS showed tendencies to be lower regulated in COVID-19 patients. Increased DNA damage might lead to the activation of NF-κB which is involved in the regulations of genes of proinflammatory cytokines that influence immune response, inflammation but also oxidative stress. The transcription factor NF-κB is activated during viral infections [52], including COVID-19, and while upregulated it stimulates the production of chemokines such as monocyte CCL2 and CXCL10, which were also significantly upregulated in our study (Fig. 2). Elevated gene expressions of CCL2 are indicators for the enhanced recruitment of monocytes and macrophages during inflammation [53,54]. Further, the increased expression of the CCL2 receptor CCR2 is associated with the migration of the aforementioned cells involved in COVID-19 infection [53,55]. We could also detect significantly higher gene expression of NrF2 in COVID-19 patients, which functions as a key regulator to balance cellular redox states by promoting enzymes involved in antioxidant defense and might be induced by increased DNA damage [25]. NrF2 is known to be regulated by a Keap1-dependent signaling mechanism where Keap1 suppresses the activation of NrF2 under normal conditions which underlines the pivotal role of NrF2 and Keap1 as key regulators in the cellular antioxidant response.

Further, proteomic analyses showed that CD8A, SLAMF1, LIF-R, HGF, CCL25, NT3 were slightly but significantly different between the COVID-19 survivors and the deceased patients, exerting various physiological effects such as immune cell response, cell proliferation, immune cell migration or development of the nervous system. However, no significant differences in the DNA damage parameters and markers for oxidative stress could be detected in the late COVID-19 patients compared to the surviving hospital COVID-19 patients. These observations were also not true for older COVID-19 patients and might be explained by stronger inflammatory responses during a viral infection in younger individuals [56]. Notably, in this study we could not confirm meaningful alterations of antioxidant capacities and increased oxidative stress parameters in COVID-19 individuals. Interestingly, the average GSSG plasma levels were significantly higher in healthy controls compared to levels in patients with acute COVID-19 infections. There are several explanations for this observation in the controls (Table 2, Table 4). The controls were healthy with a normal extent of everyday physical activity which might also increase the need for balancing endogenous cellular ROS-production; thus, increasing plasma GSSG levels. Lifestyle factors such as sedentary lifestyle and physical activity influence GSSG levels by oxidizing GSH since physical exercise intensity enhances GSSG production, especially in younger adults [57] which is also influenced by individual genetic makeup [58,59]. Interestingly, overall the GSH:GSSG ratios of the COVID-19 patients were significantly higher compared to healthy controls which indicates a shift to a more prooxidative environment in the healthy non-infected counterparts. As can be seen in Table 4, younger COVID-19 patients displayed tendencies of higher average GSH and GSSG values and showed higher average GSH:GSSG ratios compared to older COVID-19 subjects indicating a more reduced oxidative state [48]. Interestingly, the GSH:GSSG ratios of the older COVID-19 patients were also comparable to those of healthy older adults published in other studies [60]. Thus, we suggest that the acute COVID-19 infections did not adversely influence the redox state in the hospitalized patients [1,20,39,40].

As can be seen in Table 2, significant differences between COVID-19 patients and healthy controls included HDL (-48%), LDL (-44%), and total cholesterol levels (-40%). In fact, the average levels of 60 mg/dL HDL in the healthy study subjects might be protective against the risk of developing CVD; whereas the lower levels of HDL of 33 mg/dL as found in the COVID-19 patients in our study might be associated with increased CVD risks [61]. However, we found higher average LDL- and total cholesterol levels (229.4 ± 46.00 mg/dL) in the healthy controls in accordance with the guidelines of the American Heart Association [61], but not in the COVID-19 patients. Interestingly, the blood glucose levels varied significantly between COVID-19 patients and the control subjects. Whereas the healthy study participants displayed normal fasting blood glucose levels; the average blood glucose level in the COVID-19 patients was 128.1 ± 68.76 mg/dL, which is within the prediabetic range according to the American Diabetes Association and the European Association for the Study of Diabetes [62]. The elevated blood glucose levels might be explained by the fact that 29% of the COVID-19 patients included in this study were T2DM patients. The higher number of T2DM patients in the COVID-19 group might also explain the lower total and LDL-cholesterol levels in the COVID-19 group since statin prescription represents a standard medication in patients with T2DM [[63], [64], [65], [66]]. It is very well known that the severity of COVID-19 infections is strongly influenced by comorbidities such as T2DM or cardiovascular disease that might lead to hospitalization during acute infections [[1], [2], [3]]. Interestingly, one retrospective study with 99 clinical COVID-19 patients found a relationship between fasting glucose levels and Sars-Cov2 duration that leads to the assumption that increased FPG levels are associated with a longer duration of SARS-CoV-2 RNA positivity in patients [67].

FRAP, which is used to assess the total antioxidant capacity, showed no differences between groups (Table 2). Interestingly, the older patients and healthy controls showed not only comparable levels of FRAP in the plasma, but also tendencies of higher average FRAP levels than the younger patients (Table 2 Supplementary). Notably, FRAP values of the deceased COVID-19 patients were also significantly higher compared to all survivors (+35%, p = 0.045). The same was true for MDA which is another popular oxidative stress marker. There were some interesting observations regarding UCB. UCB was significantly higher in healthy controls and significantly reduced levels of UCB in COVID-19 patients (Table 2 and 2 Supplementary). UCB is known to be involved in inflammatory processes. As mentioned earlier, the COVID-19 patients in this study showed severely higher hs-CRP levels confirming active inflammatory processes that might have affected UCB levels. However, studies suggest that slightly elevated levels of indirect serum bilirubin are known to exert antioxidant properties by scavenging free radicals [29]. Additionally, one study has demonstrated that lower UCB levels are associated with weaker outcomes in ICU patient with sepsis [31] and slightly elevated levels of UCB might act cytoprotective [30]. Thus, this positive effect of UCB was affected in the COVID-19 cohort, especially in the older subgroup, since UCB levels in younger COVID-19 patients as well as healthy controls were significantly higher. Interestingly, some studies reported changes related to mortality when it comes to oxidative stress markers such as OGS (serum guanine oxidized species) in a cohort of non-surviving COVID-19 patients compared to survivors of the COVID-19 infection [41]. Since oxidative stress markers were not altered in this study, we suggest that DNA damage in COVID-19 patients might be driven by inflammation. Mechanistically, the cGAS-STING pathway plays a pivotal role in the detection of foreign genetic material including viruses such as SARS-Cov-2 [[68], [69], [70]] and consequently induces immune responses. However, a prolonged activation of this pathway might enhance the risk for chronic inflammation. This can lead to constantly increased production of proinflammatory cytokines [71] which can further induce DNA instability and DNA damage response [72] and elevated cancer risk [[73], [74], [75]]. One study has shown that inflammatory proteomic pattern in COVID-19 is affected weeks after a COVID-19 infection [76] but this pattern might be transient. Since most of the cellular DNA damage can be repaired and DNA repair is diversely influenced by circadian rhythm [77], but also individual DNA repair kinetics [78] and diseases [79,80], thus, we suggest that the inflammation-induced DNA damage caused by COVID-19 infections might be temporary [81] but this should be addressed in future studies.

**Strength and Limitations of this study:** One strength of this study is that none of the study hospitalized COVID-19 participants and only one healthy control was vaccinated against COVID-19 at the timepoint of the sampling, due to the fact that vaccinations during the time of sampling were not available for everybody. Further, although all healthy controls were tested for SARS-CoV-2 antibodies, silent early COVID-19-infections have been reported in scientific literature and cannot be entirely ruled out. The ratio of males to females as well as younger to older COVID-19 patients was equally balanced, which enabled us to compare the data of both sexes and different age groups. A plethora of biomedical parameters such as blood parameters, information about diseases, allergies, and comorbidities were available for the analyses of all study subjects. However, no further information about anthropometric parameters including body weight, height, or BMI could be evaluated for COVID-19 patients during this study due to harsh lockdown restrictions during sampling.

## 5 Conclusion

This case-control study of 48 hospitalized COVID-19 patients compared to 48 healthy age- and sex-matched counterparts revealed new clinical information regarding COVID-19-infections. To our knowledge, we are the first to report that COVID-19 patients displayed reduced levels of UCB. DNA damage markers only showed tendencies to be increased in COVID-19 patients. However, we observed significantly increased DNA damage, especially FPG-sensitive sites, in younger COVID-19 patients (average age 55 years) compared to older COVID-19 patients of around 83 years. Furthermore, younger COVID-19 patients also showed slightly but significantly increased DNA damage parameters after basic treatment with lysis buffer and after challenge with H_2_O_2_ compared to healthy controls. Moreover, inflammatory markers such as hs-CRP were strongly increased, and 64 different proteomic inflammatory markers were significantly different between COVID-19 patients and their healthy counterparts due to infection-induced activated inflammatory pathways. However, inflammatory markers were not linked to age. Strikingly, in contrast to other reports, we could not confirm that the slightly elevated DNA damage was linked to or caused by adverse alterations of the redox status of COVID-19 patients, since key antioxidant parameters such as FRAP levels or oxidative stress biomarker such as MDA levels were not significantly different between healthy controls and hospitalized patients. Contrary, hospitalized COVID-19 patients showed significantly higher GSH:GSSG ratios than their healthy counterparts. Moreover, these GSH:GSSG ratios of individuals with COVID-19 were comparable to those found in healthy older adults observed in other studies. Thus, results indicate that DNA damage in younger hospitalized COVID-19 patients might be triggered by inflammatory processes and not by oxidative stress. Since alterations of proteomic inflammatory status can be reversed after COVID-19 infections, we suggest that this inflammatory-induced DNA damage in COVID-19 patients might be transient but this should be investigated in future studies with individuals that recovered from a severe COVID-19 infection after a hospitalization.

## Funding

This work was supported by the 10.13039/501100003065University of Vienna, by funding the Research Platform Active Ageing. This article is supported by the Open Access Publishing Fund of the 10.13039/501100003065University of Vienna.

## Declaration of competing interest

The authors declare that they have no known competing financial interests or personal relationships that could have appeared to influence the work reported in this paper.

## Data Availability

Data will be made available on request.
