# Supplementary material for: Age-related influence on DNA damage, proteomic inflammatory markers and oxidative stress in hospitalized COVID-19 patients compared to healthy controls
Source: Redox Biol. 2023 Oct 3;67:102914. doi: 10.1016/j.redox.2023.102914 (PMC10585323; doi:10.1016/j.redox.2023.102914)
Supplement: Multimedia component 1 [file mmc1.docx]

| **Table 1 Supplementary:**  Sex-specific differences in DNA damage and oxidative stress parameters in COVID-19 patients (males and females) | | | | | | | | |
| --- | --- | --- | --- | --- | --- | --- | --- | --- |
|  | Males | | |  | Females | | |  |
| Parameter | All | Controls | COVID-19 |  | All | Controls | COVID-19 |  |
| Subjects | n=50 | n=25 | n=25 |  | n=48 | n=24 | n=24 |  |
|  | Mean ± SD | | | p-val. | Mean ± SD | | | p-val. |
| Age (years) | 69.9±16.1 | 70.0±16.1 | 69.8±16.4 |  | 68.8±17.1 | 68.7±17.3 | 68.8±17.3 |  |
| Sex (females/males) | 0/50 | 0/25 | 0/25 |  | 48/0 | 24/0 | 24/0 |  |
| **DNA damage parameters** | | | | | | | | |
| Lysis (% Tail DNA) | 1.92±0.78 | 1.82±0.58 | 2.01±0.95 | 0.823 | 1.96±0.77 | 1.75±0.62 | 2.17±0.85 | **0.023** |
| Netto FPG (% Tail DNA) | 9.04±6.66 | 8.04±2.55 | 10.03±6.66 | 0.138 | 9.41±5.10 | 8.12±2.73 | 7.71±2.12 | 0.307 |
| H_2_O_2_ (% Tail DNA) | 11.88±2.98 | 11.32±3.04 | 12.43±2.87 | 0.109 | 11.69±2.60 | 11.02±2.70 | 12.37±2.35 | 0.376 |
| **Oxidative stress parameters** | | | | | | | | |
| FRAP (µmol/L) | 1062±263 | 1119±210 | 1005±301 | 0.079 | 964±221 | 976±241 | 917±199 | 0.241 |
| GSH (µmol/L) | 15.15±3.78 | 15.37±2.71 | 14.92±4.69 | 0.280 | 15.47±3.86 | 16.01±2.88 | 14.91±4.68 | 0.102 |
| GSSG (µmol/L) | 9.85±3.74 | 10.70±2.62 | 8.96±4.51 | **0.003** | 9.80±3.96 | 11.20±2.23 | 8.52±4.87 | **<0.001** |
| GSH/GSSG ratio | 1.66±0.53 | 1.52±0.49 | 1.80±0.55 | **0.041** | 1.75±0.53 | 1.47±0.37 | 2.20±1.65 | **0.038** |
| MDA (µmol/L) | 1.59±0.54 | 1.63±0.43 | 1.55±0.64 | 0.273 | 1.59±0.53 | 1.58±0.44 | 1.61±0.62 | 0.684 |
| **Blood parameters** | | | | | | | | |
| hs-CRP (mg/dL) | 40.44±60.70 | 2.43±2.83 | 80.05±66.89 | **<0.001** | 36.68±65.41 | 1.54±1.59 | 73.42±78.71 | **<0.001** |
| UCB (µmol/L) | 3.81±3.61 | 4.16±3.12 | 3.47±4.08 | **0.026** | 2.98±2.04 | 1.74±0.91 | 2.20±1.65 | **0.005** |
| Vit. D (ng/mL) | 21.6±11.4 | 20.1±9.7 | 23.1±12.8 | 0.580 | 21.7±11.7 | 21.72±11.14 | 21.75±12.50 | 0.423 |
|  |  |  |  |  |  |  |  |  |
| Data are presented as means±standard deviation, p-values are calculated using Mann-Whitney U test for measuring age differences, significant differences are highlighted with bold numbers. A p-value (p) of 0.05 is considered as significant. Older healthy and older COVID-19 hospital patients are age and sex-matched. FPG: formamidopyrimidine DNA glycosylase, FRAP: ferric reducing ability potential; GSH: γ-glutamyl-cysteinyl-glycine; GSSG: glutathione disulfide; MDA: malondialdehyde; hs-CRP: high sensitivity c-reactive protein; UCB: unconjugated bilirubin | | | | | | | | |

| **Table 2 Supplementary:** Sex-specific differences in DNA damage and oxidative stress parameters in young (<69 years) COVID-19 patients (males and females) | | | | | | | | |
| --- | --- | --- | --- | --- | --- | --- | --- | --- |
|  | Young males | | |  | Young females | | |  |
| Parameter | All | Controls | COVID-19 |  | All | Controls | COVID-19 |  |
| Subjects | n=26 | n=13 | n=13 |  | n=22 | n=11 | n=11 |  |
|  | Mean ± SD | | | p-val. | Mean ± SD | | | p-val. |
| Age (years) | 56.9±9.9 | 57.2±9.8 | 56.8±10.4 |  | 54.3±12.7 | 54.3±12.4 | 54.3±12.7 |  |
| Sex (Females/Males) | 0/26 | 0/13 | 0/13 |  | 0/22 | 0/11 | 0/11 |  |
| **DNA damage parameters** | | | | | | | | |
| Lysis (% Tail DNA) | 1.97 ±0.93 | 1.73 ±0.66 | 2.22±1.11 | 0.317 | 1.94 ±0.53 | 1.77 ±0.6 | 2.11±0.42 | 0.140 |
| Netto FPG (% Tail DNA) | 10.26 ±6.51 | 8.23 ±2.92 | 12.28±8.42 | 0.061 | 8.4 ±2.39 | 7.99 ±2.62 | 8.81±2.18 | 0.341 |
| H_2_O_2_ (% Tail DNA) | 11.06 ±2.27 | 10.14 ±2.33 | 11.98±11.88 | **0.026** | 12.45 ±2.8 | 12.06 ±2.85 | 12.83±2.84 | 0.622 |
| **Oxidative stress parameters** | | | | | | | | |
| FRAP (µmol/L) | 1017±258 | 1114 ±235 | 921±252 | 0.061 | 862±125 | 811±111 | 913±123 | 0.082 |
| GSH (µmol/L) | 15.82 ±4.61 | 16.22 ±2.9 | 15.40±6.06 | 0.211 | 15.07 ±2.85 | 15.76±2.98 | 14.37±2.68 | 0.158 |
| GSSG (µmol/L) | 10.82 ±4.45 | 11.47±2.27 | 10.11±6.05 | **0.029** | 8.82 ±3.07 | 10.87 ±2.24 | 6.78±2.35 | **<0.001** |
| GSH/GSSG ratio | 1.55 ±0.43 | 1.43 ±0.18 | 1.69±0.57 | 0.550 | 1.98 ±0.99 | 1.52 ±0.52 | 2.43±1.16 | **0.028** |
| MDA (µmol/L) | 1.7 ±0.61 | 1.66 ±0.49 | 1.74±0.74 | 0.858 | 1.47 ±0.4 | 1.48 ±0.38 | 1.45±0.43 | 0.974 |
| **Blood parameters** | | | | | | | | |
| hs-CRP (mg/dL) | 29.82±41.19 | 2.68 ±2.12 | 59.23±43.39 | **<0.001** | 49.57±79.28 | 1.37 ±1.59 | 103.6±89.46 | **<0.001** |
| UCB (µmol/L) | 3.14 ±1.76 | 3.69 ±1.28 | 2.58±2.04 | **0.008** | 2.8 ±2.34 | 3.76 ±2.84 | 1.74±0.91 | **0.017** |
| Vit. D (ng/mL) | 22.47 ±13.56 | 19.46±12.54 | 25.50±14.36 | 0.228 | 20.88±9.31 | 24.4 ±9.08 | 17.35±8.45 | 0.061 |
|  |  |  |  |  |  |  |  |  |
| Data are presented as means±standard deviation, p-values are calculated using Mann-Whitney U test for measuring age differences, significant differences are highlighted with bold numbers. FPG: formamidopyrimidine DNA glycosylase, FRAP: ferric reducing ability potential; GSH: γ-glutamyl-cysteinyl-glycine; GSSG: glutathione disulfide; MDA: malondialdehyde; hs-CRP: high sensitivity c-reactive protein; UCB: unconjugated bilirubin | | | | | | | | |

| **Table 3 Supplementary:** Sex-specific differences of DNA damage and oxidative stress parameters in older (≥69 years) COVID-19 patients (males and females) | | | | | | | | |
| --- | --- | --- | --- | --- | --- | --- | --- | --- |
|  | Older males | | |  | Older females | | |  |
| Parameter | All | Controls | COVID-19 |  | All | Controls | COVID-19 |  |
| Subjects | n=24 | n=12 | n=12 |  | n=24 | n=12 | n=12 |  |
|  | Mean ± SD | | | p-val. | Mean ± SD | | | p-val. |
| Age (years) | 83.9±7.4 | 83.9±7.3 | 83.9±7.4 |  | 82.1±6.9 | 82.1±17.1 | 82.1±7.1 |  |
| Sex (Females/Males) | 0/24 | 0/12 | 0/12 |  | 0/24 | 0/12 | 0/12 |  |
| **DNA damage parameters** | | | | | | | | |
| Lysis (% Tail DNA) | 1.85 ±0.59 | 1.92 ±0.47 | 1.79±0.71 | 0.248 | 1.84 ±0.45 | 1.69 ±0.45 | 2.00±0.42 | 0.097 |
| Netto FPG (% Tail DNA) | 7.72 ±2.38 | 7.84 ±2.19 | 7.59±2.64 | 1.000 | 7.54 ±1.85 | 7.34 ±1.62 | 7.75±2.14 | 0.854 |
| H_2_O_2_ (% Tail DNA) | 12.76 ±3.42 | 12.6 ±3.29 | 12.93±3.69 | 0.908 | 11.58 ±2.83 | 11.14 ±2.45 | 12.06±3.24 | 0.580 |
| **Oxidative stress parameters** | | | | | | | | |
| FRAP (µmol/L) | 1109±264 | 1123±187 | 1096±333 | 0.644 | 863±216 | 841 ±141 | 885±279 | 0.974 |
| GSH (µmol/L) | 14.45 ±2.58 | 14.46±2.25 | 14.44±2.98 | 0.817 | 13.63 ±2.73 | 14.08 ±3.42 | 13.81±1.84 | 0.488 |
| GSSG (µmol/L) | 8.83 ±2.52 | 9.86 ±2.81 | 7.80±1.74 | 0.053 | 8.34 ±2.72 | 8.79 ±2.38 | 7.88±3.05 | 0.194 |
| GSH/GSSG ratio | 1.77 ±0.61 | 1.62 ±0.68 | 1.92±0.52 | 0.106 | 1.8 ±0.65 | 1.74 ±0.66 | 1.85±0.66 | 0.488 |
| MDA (µmol/L) | 1.47 ±0.43 | 1.60 ±0.37 | 1.35±0.46 | 0.166 | 1.55 ±0.49 | 1.44 ±0.35 | 1.65±0.58 | 0.525 |
| **Blood parameters** | | | | | | | | |
| hs-CRP (mg/dL) | 51.51±75.29 | 2.16 ±3.53 | 100.87±80.79 | **<0.001** | 25.4±49.35 | 1.69 ±1.65 | 49.11±62.16 | **<0.001** |
| UCB (µmol/L) | 4.63 ±4.94 | 4.71 ±4.47 | 4.54±5.61 | 1.000 | 2.37 ±1.72 | 3.07 ±2.06 | 1.66±0.94 | 0.165 |
| Vit. D (ng/mL) | 20.62 ±8.52 | 20.77 ±5.89 | 20.48±10.82 | 0.644 | 25.83 ±11 | 25.23±7.44 | 26.43±14.02 | 0.644 |
|  |  |  |  |  |  |  |  |  |
| Data are presented as means±standard deviation, p-values are calculated using Mann-Whitney U test for measuring age differences, significant differences are highlighted with bold numbers. FPG: formamidopyrimidine DNA glycosylase, FRAP: ferric reducing ability potential; GSH: γ-glutamyl-cysteinyl-glycine; GSSG: glutathione disulfide; MDA: malondialdehyde; hs-CRP: high sensitivity c-reactive protein; UCB: unconjugated bilirubin   \| **Table 4 Supplementary:** Parameters of COVID-19 hospital patients divided by age: 69 years \| \| \| \| \| \| \| \| --- \| --- \| --- \| --- \| --- \| --- \| --- \| \|  \| COVID-19 patients \| \| \| Healthy controls \| \| \| Parameter \| Older  (≥ 69 y.) \| Younger  (< 69 y.) \|  \| Older  (≥ 69 y.) \| Younger  (< 69 y.) \|  \| \| Subjects \| n=24 \| n=24 \| p-value \| n=24 \| n=24 \| p-value \| \| **DNA damage parameters** \| \| \| \| \| \| \| \| Lysis (% Tail DNA) \| 1.89±0.60 \| 2.17±0.85 \| 0.197 \| 1.80±0.46 \| 1.75±0.62 \| 0.730 \| \| Netto FPG (% Tail DNA) \| 7.67±2.36 \| 10.69±6.50 \| **0.041** \| 7.59±1.90 \| 8.12±2.73 \| 0.442 \| \| H_2_O_2_ (% Tail DNA) \| 12.51±3.43 \| 12.37±2.35 \| 0.869 \| 11.87±2.93 \| 11.02±2.70 \| 0.301 \| \| **Oxidative stress parameters** \| \| \| \| \| \| \| \| FRAP (µmol/L) \| 995±319 \| 917±199 \| 0.326 \| 989±218 \| 976±241 \| 0.846 \| \| GSH (µmol/L) \| 13.81±2.50 \| 14.91±4.68 \| 0.327 \| 14.27±2.84 \| 16.01±2.88 \| **0.041** \| \| GSSG (µmol/L) \| 7.84±2.43 \| 8.52±4.87 \| 0.554 \| 9.33±2.60 \| 11.20±2.23 \| **0.010** \| \| GSH/GSSG ratio \| 1.89±0.59 \| 2.20±1.65 \| 0.508 \| 1.68±0.66 \| 1.47±0.37 \| 0.193 \| \| MDA (µmol/L) \| 1.50±0.54 \| 1.61±0.62 \| 0.528 \| 1.52±0.36 \| 1.58±0.44 \| 0.610 \| \| **Blood parameters** \| \| \| \| \| \| \| \| hs-CRP (mg/L) \| 74.99±75.28 \| 78.94±70.03 \| 0.855 \| 1.93±2.70 \| 2.08±1,97 \| 0.823 \| \| UCB (µmol/L) \| 3.10±4.18 \| 2.20±1.65 \| 0.374 \| 3.89±3.49 \| 3.72±2.12 \| 0.852 \| \| Vitamin D (ng/ml) \| 23.45±12.62 \| 21.75±12.50 \| 0.642 \| 23.00±6.95 \| 21.72±11.14 \| 0.637 \| \| Data are presented as means ± standard deviation, p-values are calculated using Mann-Whitney U test for measuring age differences, significant differences are highlighted with bold numbers. A p-value (p) of 0.05 is considered as significant. Older healthy and older COVID-19 hospital patients are age and sex-matched. FPG: formamidopyrimidine DNA glycosylase, FRAP: ferric reducing ability potential; GSH: γ-glutamyl-cysteinyl-glycine; GSSG: glutathione disulfide; MDA. malondialdehyde; hs-CRP: high sensitivity c-reactive protein; ALAT: alanine-aminotransferase; ASAT: aspartate-aminotransferase, UCB: unconjugated bilirubin; HDL: high density lipoprotein; LDL: low density lipoprotein. \| \| \| \| \| \| \| | | | | | | | | |

**B**

**C**

**A**

**Figure 1 Supplementary**: **A**. Dot plot presentation showing the relative expression of mRNA levels of CCL2 in COVID-19 patients (left) and healthy controls (HC) (right). **B.** Dot plot presentation of the CCL2 gene in COVID-19 males vs. healthy males. **C.** Dot plot of CCL2 gene expression in COVID-19 females vs. healthy females. Whiskers are showing the standard error of the mean. The asterisk (* ) indicates statistically significant difference; *p<0.05. **A.** The ΔCt values of the CCL2 gene of all COVID-19 patients enrolled in the ABCD study compared with healthy controls (HC). **B**. A broad distribution of the ΔCt values of the CCL2 gene can be seen male COVID-19 patients, compared to the ΔCt of the CCL2 gene of the healthy controls. **C**. Significantly different CCL2 gene expression have been measured in female COVID-19 patients; *p<0.05.


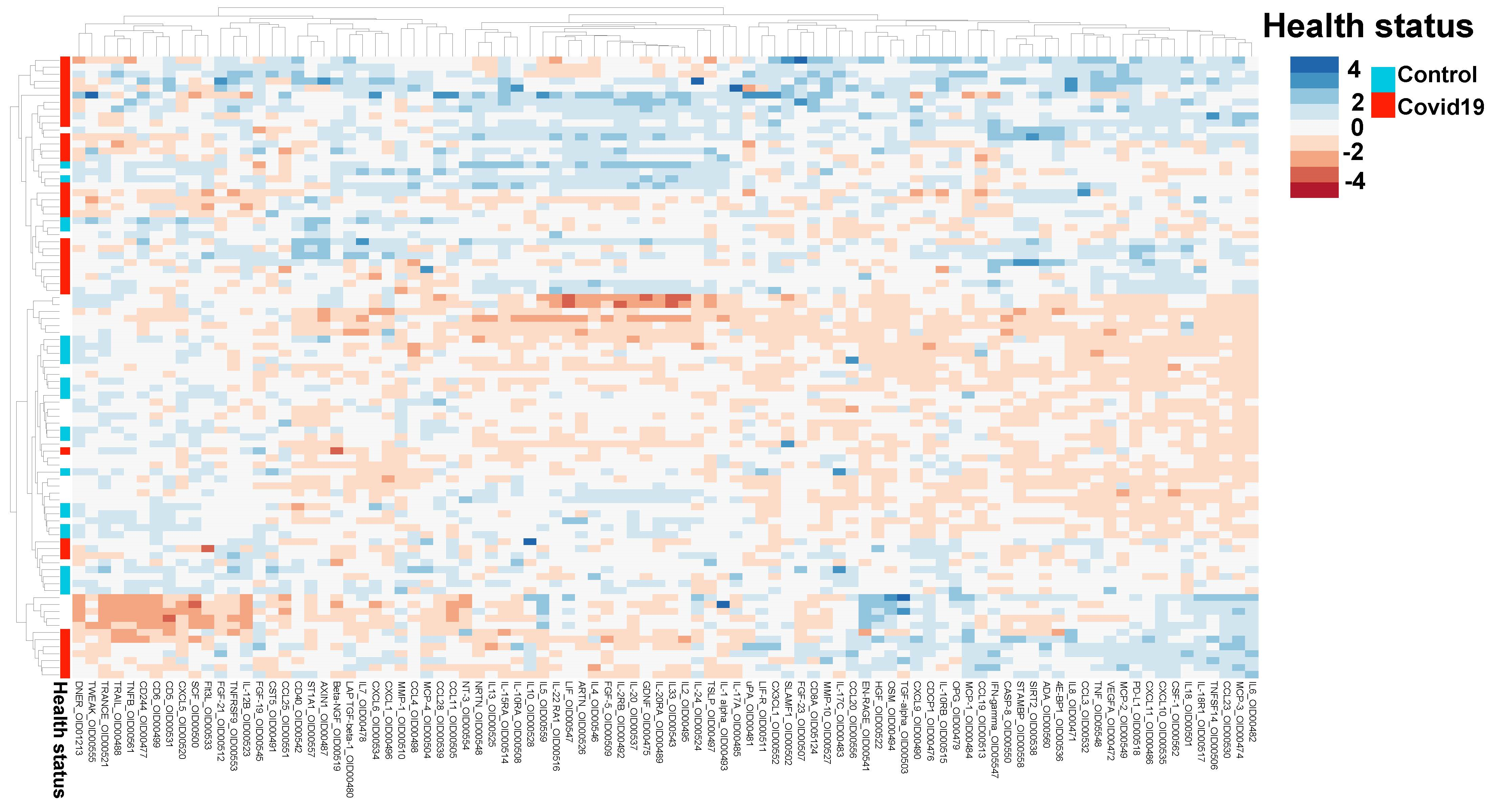


**Figure 2 Supplementary:** The heatmap shows 64 significantly differentially abundant proteins with opposite expression patterns in COVID-19 patients vs. healthy controls. Of a total of 76 proteins that were analyzed, 55 were significantly upregulated whereas 9 were significantly downregulated in individuals with COVID-19 compared to healthy controls. Columns represent each protein analyzed and rows are showing each sample analyzed. The bar on the left indicates health status. Z-score scaling are depicted across the columns.
